# A real-world study of effectiveness of intravitreal bevacizumab and ranibizumab injection for treating retinal diseases in Thailand

**DOI:** 10.1186/s12886-019-1086-1

**Published:** 2019-03-29

**Authors:** Suthasinee Kumluang, Lily Ingsrisawang, Sermsiri Sangroongruangsri, Usa Chaikledkaew, Tanapat Ratanapakorn, Paisan Ruamviboonsuk, Wongsiri Taweebanjongsin, Janejit Choovuthayakorn, Somanus Thoongsuwan, Prut Hanutsaha, Kittisak Kulvichit, Thitiporn Ratanapojnard, Warapat Wongsawad, Pattara Leelahavarong, Yot Teerawattananon

**Affiliations:** 1 Health Intervention and Technology Assessment Program (HITAP), Ministry of Public Health, Nonthaburi, Thailand; 2 Department of Statistics, Faculty of Science, Kasetsart University, Bangkok, Thailand; 3 Social and Administrative Pharmacy Excellence Research (SAPER) Unit, Department of Pharmacy, Faculty of Pharmacy, Mahidol University, Bangkok, Thailand; 4 Department of Ophthalmology, Faculty of Medicine, Khon Kaen University, Khon Kaen, Thailand; 5 Department of Ophthalmology, Rajavithi Hospital, Bangkok, Thailand; 6 Department of Ophthalmology, Mettapracharak (Wat Rai Khing) Hospital, Nakornprathom, Thailand; 7 Department of Ophthalmology, Faculty of Medicine, Chiang Mai University, Chiang Mai, Thailand; 8 Department of Ophthalmology, Faculty of Medicine, Siriraj Hospital, Mahidol University, Bangkok, Thailand; 9 Department of Ophthalmology, Faculty of Medicine, Ramathibodi Hospital, Mahidol University, Bangkok, Thailand; 10 Vitreo-Retina Research Unit, Department of Ophthalmology, Faculty of Medicine, Chulalongkorn University, Bangkok, Thailand; 11 Department of Ophthalmology, Phramongkutklao Hospital, Phramongkutklao College of Medicine, Bangkok, Thailand

**Keywords:** Bevacizumab, Ranibizumab, Retinal diseases, Real world, Vision improvement

## Abstract

**Background:**

To evaluate the effectiveness of intravitreal bevacizumab (IVB) and intravitreal ranibizumab (IVR) in actual practice for treating patients with retinal diseases in Thailand.

**Methods:**

A prospective, multi-centre, observational study was conducted among eight hospitals in their ophthalmology outpatient departments. Participants consisted of patients who had previously not received any IVB or IVR treatment between 2013 and 2014. The primary outcome measurement was the change in best-corrected visual acuity (BCVA) at the end of the follow-up period compared to baseline.

**Results:**

There were 1629 treatment-naïve patients for the pro re nata (PRN) treatment pattern and 226 treatment-naive patients for the three-injections (3Inj) treatment pattern. BCVA improvements were found in 35% of the PRN group and 47% of the 3Inj group; however, it was not clinically meaningful between the IVB and IVR groups (*P*-value = 0.568 for PRN, P-value = 0.103 for 3Inj). A multivariable logistic regression (using the propensity score) showed that positive factors associated with vision improvement for the PRN pattern were the number of drug injections, having retinal vein occlusion, and under 60 years of age, while good BCVA at baseline was a negative predictive factor. For the 3Inj pattern, under 60 years of age and baseline BCVA were statistically significant predictors. Nonetheless, diabetes mellitus (DM) without other comorbidities was a statistically significant predictor of low response to vision improvement compared to DM with other comorbidities.

**Conclusions:**

This study was the first observational, prospective study to evaluate the real-life effectiveness of IVB and IVR in Thailand. The majority of participants who used IVB or IVR showed improvements in BCVA after treatment. Further evaluation such as long-term follow-ups and subsequent comparison of effectiveness between IVB and IVR should be investigated due to the limited sample of IVR patients.

**Trial registration:**

Thai Clinical Trial Registry TCTR20141002001. Registered 02 October 2014 (retrospectively registered).

## Background

Neovascular age-related macular degeneration (nAMD), diabetic macular edema (DME), and retinal vein occlusion (RVO) are well-recognised as the most common causes of visual impairment and blindness in elderly populations [1–4]. Population-based studies have highlighted the global prevalence of these diseases, with the pooled prevalence of AMD and late AMD at 8.69 and 0.37%, respectively [5], DME prevalence at 7% [2, 6, 7], and age- and sex-standardised RVO prevalence at 0.5%.

These retinal diseases are also major eye problems in Thailand. The nAMD epidemiological cross-sectional survey in 2010 revealed that the prevalence rate of AMD among the Thai population over 50 years of age was 12.2%; 0.7% of that figure was nAMD [8]. The prevalence of RVO was reported to be between 0.5 and 2% of the total population [1], and DME prevalence was found to be between 2 and 3% in Thai diabetic patients [2, 9].

Vascular endothelial growth factor (VEGF) plays a critical role in vision loss due to angiogenesis and vascular hyperpermeability. Anti-VEGF drugs such as bevacizumab (off-label) and ranibizumab (licensed) have been widely used by ophthalmologists for treating nAMD, DME, and RVO since 2006 [10, 11]. Although numerous studies have shown that both drugs are beneficial and comparatively effective for these retinal diseases [12, 13], price differences between the two medications have driven the use of off-label intravitreal bevacizumab (IVB) as a less expensive alternative, with many countries experiencing good evidence-based results [14–16].

In 2012, a systematic review and meta-analysis conducted by the Health Intervention and Technology Assessment Program (HITAP) [9] indicated that IVB was superior to non-pharmaceutical interventions in nAMD and DME. The results also found that clinical outcomes such as improving Visual Acuity (VA), reducing Central Macular Thickness (CMT), and treatment responses between IVB and intravitreal ranibizumab (IVR) were indifferent for both diseases. Finally, after reporting the results to the Sub-committee for the Development of the National List of Essential Medicines (NLEM) of Thailand, the Sub-committee approved the inclusion of bevacizumab into the NLEM for nAMD and DME despite its off-label status to address the issues of anti-VEGF drug affordability and IVB treatment accessibility. This was because the three major public health insurance schemes (HIS) in Thailand - which reference the NLEM as the basic package for drug benefits - covers the majority of the Thai population; the Civil Servant Medical Benefit Scheme (CSMBS) covers 9%, the Social Security Scheme (SSS) covers 16%, and the Universal Coverage Scheme (UCS) covers 75% [17–19]. However, certain drugs on the non-essential drug (NED) lists - including ranibizumab - can only be reimbursed by those under the CSMBS while the other two schemes require out-of-pocket payments. For this reason, not only does it contribute to differences in services provided but it also results in inequitable access to essential medicines - contradicting the notion that access to essential health interventions is considered a basic right for patients.

The purpose of this study is to analyse the clinical outcomes of IVB and IVR for treating retinal diseases using real-world data in Thailand. The evidence generated from this study will provide information on whether IVB should be used in macular diseases. It is also expected that the results of this study will be used to inform the NLEM Sub-committee about further optimisations, as well as to provide a reference for decision-makers in other developing countries with similar interests.

## Methods

### Participant and study design

This study was part of a main study which focuses on the safety of bevacizumab in real-world settings. The parent study was an observational, non-interventional, multi-centre, prospective study and was conducted in 8 Thai tertiary and teaching hospitals between January 2013 and August 2014 [20]. In both this study and the parent study, participants underwent comprehensive ophthalmologic examinations and treatments based on normal clinical practices. Treatment plans or decisions were left to the clinical and discretionary judgment of the treating retina specialist over the follow-up period.

Inclusion criteria for this study included retinal disease patients who had not been previously treated with either IVB (1.25 mg/0.05 ml) or IVR (0.5 mg/0.05 ml), were over 18 years old, and did not switch between IVB and IVR upon receiving treatment during the study period (6 months). Exclusion criteria consisted of any history with previous IVB or IVR injections and switching of treatment to something other than an intravitreal VEGF inhibitor. Prior to data collection, informed written consent was obtained from each patient.

### Interventions

This study followed routine clinical practice. Decisions about eligibility and anti-VEGF drug selection (1.25 mg of IVB or 0.5 mg of IVR) were based on the clinical and discretionary judgment of the ophthalmologists, and these decisions were aligned with the retinal treatment guideline [21] and current NLEM treatment guidelines for the usage and monitoring of bevacizumab for nAMD and DME patients [22, 23]; they were also consistent with the two previous landmark RCTs [24, 25].

In this study, the interventions of interest were classified into two groups: 1) an initial treatment course called the three-injections pattern (3Inj) – also known as the standard regimen – which served as the loading phase of three consecutive intravitreal (IVB or IVR) monthly injections as recommended in the NLEM’s criteria [22], and 2) IVB or IVR injections as needed (pro re nata or PRN). These groups were specifically chosen to determine the differences in IVB and IVR for both the NLEM recommendation (the 3Inj pattern) and actual clinical practices (the PRN pattern). To determine VA improvement, BCVA was initially measured prior to any injections to serve as a baseline, and subsequent BCVA measurements in both groups were conducted within 28–35 days after each injection using identical measurement methods [22]. Therefore, BCVA was measured a total of 4 times for the 3Inj group (the 4th BCVA measurement occurred within 35 days after the 3rd injection). However, there was no fixed amount of BCVA measurements for the PRN group as the number of injections depended on the ophthalmologist’s prescription.

In terms of classifying patients into the two treatment groups, patients who were prescribed the 3Inj treatment course were considered 3Inj samples. However, to be classified into the PRN treatment group, DME patients needed to have a minimum of 1 anti-VEGF injection but not more than 6 injections per year and nAMD patients needed to have a minimum of 1 anti-VEGF injection but not more than 12 injections per year [22]. Moreover, the NLEM recommends that patients who receive more than 3 injections should automatically be classified into the PRN treatment. For example, if the patient received 6 injections in total, he/she would be considered a sample in both 3Inj and PRN groups. The difference is that the number of injections used for the effective analysis in the 3Inj group would be 3, while the number of injections would be 6 for the PRN pattern group. The reason for this is that the authors would like to know the effectiveness of the drug in its loading phase (3 injections) as well as its effectiveness in actual practice. Subsequently, a data analysis was performed one at a time for each treatment pattern group.

### Data collection

The data collection process comprised patient interviews and medical record reviews. Data quality was carefully managed by training the research staff to interview patients, review medical records, and extract patient data in the designed paper case report forms (pCRFs). Retinal specialists were responsible for verifying the medical data as well as the pCRFs. Once the data were documented in the pCRFs by the first research staff, the pCRFs were randomly reviewed and corrected by the second research staff to ensure accuracy and completeness. Afterwards, an independent double data entry and validation process for the pCRFs was conducted using the Epidata® programme. Prior to analysis, the data discrepancy in the database was verified and reconciled – especially the baseline data of participants and the diagnosis or other clinical data – and then compared to the original pCRFs or medical records. If necessary, a third party, such as a retina specialist, was used to confirm the accuracy of the data.

The collected data consisted of demographic and baseline characteristics and information from reviewed medical records for treatments such as ophthalmologic diagnoses, retinal examinations, VA scores, and anti-VEGF drug used. Demographic and baseline characteristics were recorded during the initial visit comprised age, sex, comorbid conditions, and type of health insurance. These characteristics were updated in follow-up visits, and these were scheduled within 35 days of the current visit; the overall duration for tracking follow-up sessions was 6 months.

### Outcome measures

To measure visual function, VA or BCVA was measured on the eye to be treated according to the method used in routine care - by using a patient’s eyeglasses or a pinhole. If both eyes were equally qualified, one eye would be chosen at random. The measurement methods comprised four steps from least severe to most severe (5 levels of severity) as follows: 1) Check whether the patient can read a chart. If yes, then use either the Snellen or ETDRS chart depending on the provider’s current practice. Otherwise, apply the counting fingers (CF) method; 2) If the patient is unable to count fingers, then apply the hand movements (HM) method; 3) If the patient is unable to see hand movements, then apply the perception of light (PL) method; 4) Ask the patient whether he/she can detect light. If yes, then the patient is classified as able to perceive light. Otherwise, he/she is classified as no perception of light (NPL).

**Fig. 1:**
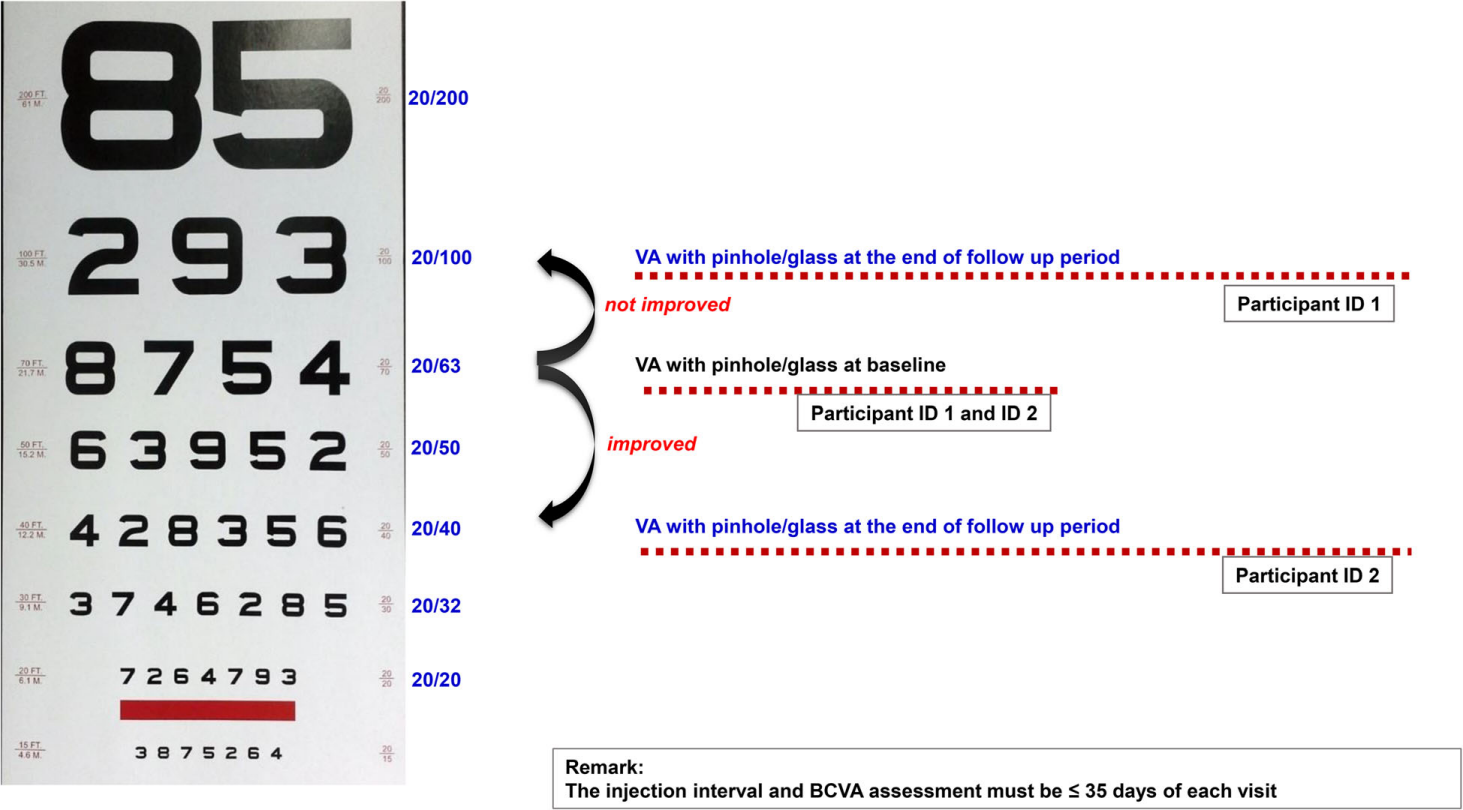
Effectiveness analysis and outcome assessment

Medical data of every patient visit were extracted from medical records. However, CMT measurements were not included as they are optional in Thailand. Due to this reason, the effectiveness analysis included only datasets of patients who had at least one intravitreal anti-VEGF injection and a post-baseline VA assessment on the treated eye; the post-baseline VA assessment must have been measured within 35 days after the previous visit using the same measurement method. For example, if a patient was tested using the pinhole method for their baseline measurement, he/she must be measured by the pinhole method for all subsequent visits until the end of the follow-up period (Fig. 1).

The primary outcome was the proportion of patients who had VA gains of more than two lines (approximately 10 ETDRS letters) at the end of the follow-up period (6 months) compared to the baseline; the secondary outcome was the associated factors that contributed to visual improvement. For the primary outcome, a novel method proposed by Gregori et al. [26] was used to convert VA measured by the Snellen chart into approximate EDTRS letter scores in order to simplify the statistical analysis. The equation used is as the following equation:

Approximate ETDRS letters = 85 + 50 × log (Snellen fraction).

In terms of outcomes, this study differed from previous clinical trials or retrospective studies such as the Comparison of Age-related macular degeneration Treatments Trials (CATT) and the Inhibition of VEGF in Age-related choroidal Neovascularisation (IVAN) trials. The difference was that those studies included only participants who could read the eye chart. However, in the Thai context, anti-VEGF drugs were prescribed even though patients failed to read the eye chart. If VA was at least two Snellen/ETDRS lines better compared to the baseline or previous visit, it was acceptable in practice as a stable or good clinical response. Additionally, this study assumed that patients who could not read the eye chart showed VA improvement if their VA measurement classification decreased in terms of severity, e.g. VA improved if the baseline VA was measured using PL and the VA method used at the 6-month follow-up was HM.

### Statistical analysis

Demographic characteristics and changes in BCVA and CMT at the baseline and last visit at the end of follow-up period were described in terms of frequencies, percentages, means and standard deviations (SD) or median and interquartile range (IQR). The Mann–Whitney U test or the Wilcoxon signed-rank test was performed for non-normally distributed variables. A dependent t-test for normally distributed variables was used for continuous variables including the change in VA between the baseline and the end of follow-up period. Frequencies, percentages, the Chi-Square test or Fisher’s exact test were used for categorical variables. Univariate and multivariable logistic regression analyses with the propensity score weighting-adjusted method were applied when evaluating the effect of potential variables on vision improvement in order to reduce the effects of selection bias and confounding [27, 28]. A two-sided probability value (*P*-value) < 0.05 was considered statistically significant. The analyses were performed using Stata12 (StataCorp LP.: Stata Statistical Software: Release 12, College Station, TX, USA).

## Results

### Characteristics of the participants

Among the 3257 anti-VEGF-naive patients included in the effectiveness cohort, 3097 patients received IVB injections (95%) and 160 patients received IVR injections (5%). The majority of patients in the IVB group was female while the majority of patients in the IVR group was male. The median age for the IVB group was 57 years (IQR 15, 50–65) and it was 63 years (IQR 19, 54–73) for the IVR group. In terms of the Thai health care schemes, more than 75% of the IVB group were covered under the UCS and SSS whereas only 14% of the IVB group were covered under the CSMBS. In contrast, nearly 38% of the IVR group were covered under the CSMBS while the percentage of non-CSMBS patients - including those under the UCS and SSS - decreased to 42%. The top three comorbidities for both groups of participants were diabetes mellitus (DM), hypertension (HTN), and dyslipidaemia (DLP). The most common diagnosis in the IVB group was DME while nAMD and polypoidal choroidal vasculopathy (PCV) were the most common diagnoses in the IVR group. Median BCVA at the baseline was approximately 50 letters (IQR 30.10, 35–65.10) or 20/100 for the IVB group and 55 letters (IQR 30.10, 35–65.10) or 20/80 for the IVR group. Therefore, baseline visual function was similar between the two groups. Baseline characteristics for all participants are shown in Table 1.

**Table 1:** Baseline characteristics of patients

Variables	IVB (*n* = 3097)	IVR (*n* = 160)	*P*-value
	n (%)	n (%)	
Gender			0.008*
Female	1670 (54)	69 (43)	
Male	1427 (46)	91 (57)	
Age			0.000*
Median (IQR; range)	57 (15; 50–65)	63 (19; 54–73)	
18–50 years	847 (27)	33 (20)	
51–60 years	1037 (34)	30 (19)	
> 60 years	1200 (39)	97 (61)	
Universal health coverage			0.000*
CSMBS	432 (14)	60 (38)	
Non-CSMBS^a^	2358 (76)	67 (42)	
Smoking history			0.500
No	2796 (90)	142 (89)	
Yes	298 (10)	18 (11)	
Underlying Diseases			
Diabetes mellitus	2177 (70)	74 (46)	0.000*
Hypertension	2047 (66)	99 (62)	0.262
Dyslipidaemia	1618 (52)	84 (53)	0.960
Chronic kidney disease	362 (12)	12 (8)	0.104
Ischaemic heart disease	185 (6)	10 (6)	0.889
Stroke	120 (4)	15 (9)	0.001*
Treated eye			0.001*
One eye (right or left eye)	2537 (82)	148 (92)	
Both eyes	560 (18)	12 (8)	
Retinal disease^b^			0.000*
nAMD and PCV	452 (12)	52 (30)	
Diabetic macular edema (DME)	1293 (35)	21 (12)	
Retinal vein occlusion (RVO)	461 (13)	47 (27)	
Proliferative diabetic retinopathy (PDR)	963 (26)	26 (15)	
Others^c^	484 (13)	25 (15)	
Visual Acuity (VA) at baseline^b^ (approximate Snellen equivalent)			0.195
Reading from Snellen/ETDRS chart	2427 (66)	105 (61)	
Median VA (IQR; range)	50.05 (20/100) (30.10, 35–65.10)	54.90 (20/80) (30.10, 35–65.10)	0.6474
CF, HM, PL, and NPL	1179 (32)	63 (37)	

^a^Includes Universal Coverage Scheme (UCS) and Social Security Scheme (SSS)

^b^From the treated eye at baseline (unit: eye)

^c^Other disorders such as vitreous haemorrhage, subretinal haemorrhage, and subretinal fluid

*P-value < 0.05

*CF* Counting fingersm, *CSMBS* Civil Servant Medical Benefit Scheme, *ETDRS* Early Treatment Diabetic Retinopathy Study, *HM* Hand movements, *IQR* interquartile range, *nAMD* Neovascular age-related macular degeneration, *NPL* No perception of light, *n* Number of patients, *PCV* Polypoidal choroidal vasculopathy, *PL* Perception of light

### VA outcomes

To conduct the effectiveness analysis, only one eye was tested per patient. For those who were treated for both eyes, one eye was randomly selected to be included in the analysis. In total, there were 1629 PRN patients and 226 3Inj patients that used the same VA measurement for all visits during the follow-up period. Out of the 1629 patients who underwent the PRN treatment pattern, 1542 patients (95%) were from the IVB group and 87 patients (5%) were from the IVR group.

In terms of changes in VA, only 567 (35%) patients attained visual improvement equal to or more than 10 ETDRS letters; 535 of these patients (35%) were from the IVB group and 32 from the IVR group (37%). Moreover, the 226 3Inj patients - comprising 201 IVB group patients and 25 IVR group patients – were also classified as a subset of the PRN pattern based on the NLEM guidelines described earlier. The change in VA outcomes showed that there were only 107 patients (47%) - comprising 99 IVB patients (49%) and 8 IVR patients (32%) - who showed visual improvement equal to or more than 10 ETDRS letters. The findings from both patterns revealed that vision improvement was not statistically significant different between the IVB and IVR groups (PRN pattern: *p* = 0.568 and 3Inj pattern: *p* = 0.103).

### Change in VA letters between baseline and the last follow-up period during the 6-month study period (baseline/3Inj and baseline/PRN)

Patients who showed VA improvement at the end of the 6-month period for each drug were classified into five groups based on letter count at the baseline. VA scores were classified into ranges as follows: 1) VA score of 68–85 letters; 2) VA score of 53–67 letters; 3) VA score of 38–52 letters; 4) VA score of 23–37 letters; and 5) VA score of 0–22 letters. A significant improvement in ETDRS letters was detected among all patients (Table 2). Improvement in the median ETDRS letters was seen in the PRN group for both IVB and IVR patients; the lone exception where improvement was not seen was for IVR patients who recorded more than 53 ETDRS letters at the baseline. In the follow-up period for the 3Inj pattern, there was a significant improvement in patients who had lower than 67 ETDRS letters in the IVB group but no significant difference in VA scores in the entire IVR group.

**Table 2:** Median ETDRS letters for the PRN pattern and 3Inj pattern

ETDRS letters	IVB		n	*P*-value	IVR		n	*P*-value
	Median baseline VA (IQR)	Median VA at 6 months (IQR)			Median baseline VA (IQR)	Median VA at 6 months (IQR)		
The PRN pattern								
68–85 letters	69.95 (69.95–76.20)	85.00 (80.15–85.00)	13	0.0013*	69.95 (69.95–69.95)	85.00 (85.00–85.00)	1	**
53–67 letters	57.80 (54.90–61.14)	69.95 (69.95–76.20)	84	0.0000*	56.35 (54.90–58.88)	69.95 (67.52–69.95)	4	0.0679
38–52 letters	46.09 (41.25–50.05)	60.08 (54.90–65.10)	98	0.0000*	46.09 (43.67–50.05)	62.54 (57.80–69.95)	8	0.0116*
23–37 letters	35.00 (35.00–35.00)	54.90 (50.15–65.10)	99	0.0000*	35.00 (29.87–35.00)	54.90 (50.05–65.10)	6	0.0277*
0–22 letters	0.00 (0.00–0.00)	35.00 (19.95–54.90)	241	0.0000*	0.00 (0.00–4.90)	57.80 (57.80–59.97)	13	0.0016*
The 3Inj pattern								
68–85 letters	76.20 (69.95–76.20)	85.00 (85.00–85.00)	3	0.1025	69.95 (69.95–69.95)	85.00 (85.00–85.00)	1	**
53–67 letters	60.08 (54.90–61.14)	69.95 (69.95–80.15)	15	0.0006*	59.97 (59.97–59.97)	69.95 (69.95–69.95)	1	**
38–52 letters	46.09 (45.21–50.05)	65.10 (60.08–69.95)	22	0.0000*	46.09 (46.09–46.09)	65.10 (65.10–65.10)	1	**
23–37 letters	35.00 (35.00–35.00)	60.03 (54.90–69.95))	37	0.0000*	32.43 (29.87–35.00)	67.53 (65.10–69.95)	2	0.1797
0–22 letters	0.00 (0.00–0.00)	50.05 (35.00–57.80)	1	**	0.00 (0.00–0.00)	65.10 (0.00–69.95)	3	0.1655

* *P*-value < 0.05, ** Not enough samples to test for VA improvement

*ETDRS* Early Treatment Diabetic Retinopathy Study; *IQR* Interquartile range; *n* Number of patients; *VA* Visual Acuity

### Diagnosis and treatment

Diagnosis of retinal diseases in the study eye is summarized in Table 3. Among the patients who received IVB, the highest percentage of patients consisted of RVO patients in both the PRN (57%) and 3Inj (63%) groups. Compared to the proportion of RVO patients, the proportion of patients diagnosed with nAMD and PCV and those diagnosed with DME showed low VA improvement. For the PRN group, there was a 36% improvement in nAMD and PCV patients and 31% improvement in DME patients, while the 3Inj group recorded a 46% improvement for both nAMD and PCV patients and DME patients.

**Table 3:** Diagnosis of retinal diseases in the study eye

	IVB			IVR		
	Number of diagnosed patients	Number of VA improved patients		Number of diagnosed patients	Number of VA improved patients	
		n	%		n	%
The PRN pattern						
nAMD and PCV	228	81	36	25	8	32
DME	464	145	31	10	3	30
RVO	241	137	57	26	12	46
PDR	389	117	30	12	4	33
Others^*^	220	55	25	14	5	36
total	1542	535	35	87	32	37
The 3Inj pattern						
nAMD and PCV	57	26	46	12	4	33
DME	52	24	46	1	0	0
RVO	52	33	63	4	1	25
PDR	15	5	33	1	1	100
Others^*^	25	11	44	7	2	29
total	201	99	49	25	8	32

*Other disorders such as vitreous haemorrhage, subretinal haemorrhage, and subretinal fluid

*DME* Diabetic macular edema; *nAMD* Neovascular age-related macular degeneration; *n*, Number of patients; *PCV* Polypoidal choroidal vasculopathy; *RVO* Retinal vein occlusion; PDR, Proliferative diabetic retinopathy

For patients treated with IVR, RVO patients comprised the highest percentage in the PRN group, same as the IVB patients. Out of the 25 IVR patients who were part of the 3Inj group, 8 showed VA improvement (32%), and 4 of the 12 patients diagnosed with nAMD and PCV showed VA improvement as well. Therefore, the findings showed that both drugs were able to noticeably improve visual function in RVO patients (57% in IVB and 46% in IVR) in the PRN group.

### Number of injections

**Fig. 2:**
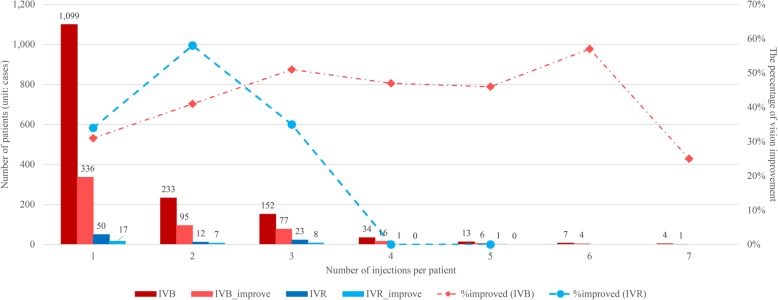
Total injections per patient and percentage of vision improvement. The bar chart represents the number of patients (left y-axis) relative to the number of injections per patient (x-axis). The percentages of vision improvement (right y-axis) relative to each number of injections are indicated as line graphs

According to the effectiveness results for the PRN group, most patients received a single injection and measured VA measurement during the follow-up period of not more than 35 days from the injection date (71% of IVB patients and 57% of IVR patients - Fig. 2). As shown, the percentage of vision improvement for IVB patients increased noticeably among those who were treated with one to three injections.

**Fig. 3:**
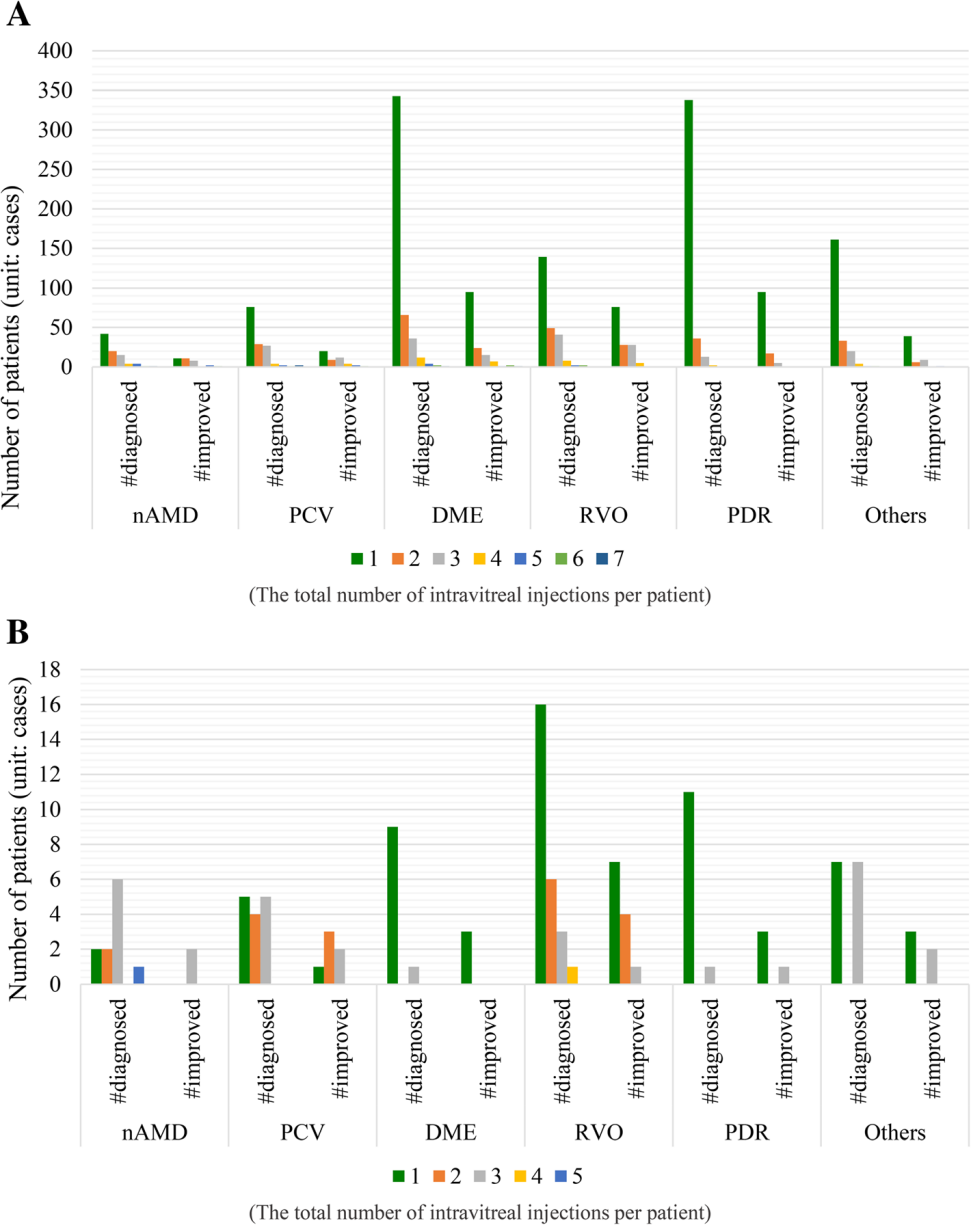
Number of patients who recorded improved VA scores for each eye disease. **a** Patients who received IVB. **b** Patients who received IVR. The numbers 1 to 7 and 1 to 5 represent the total number of IVB and IVR injections per patient for each eye disease

The results also showed that based on the retinal diseases and the number of patients who experienced VA improvement, RVO patients represented the highest proportion of those with improved VA at approximately 57 and 46% for the IVB and IVR groups, respectively, when classified by the total number of intravitreal injections per patient in the PRN pattern (Fig. 3).

### Logistic regression

Table 4 presents the univariate and propensity score-adjusted multivariable analyses for the association of potential factors affecting VA improvement from baseline and at the end of follow-up period for both PRN and 3Inj groups. In the univariate analysis of the PRN group, non - DM with and without other comorbidities, disease diagnosis, baseline VA score, and number of drug injections were significantly associated with vision improvement (VA gain ≥10 ETDRS letters at the end of follow-up period compared to baseline VA). However, in the 3Inj pattern, it was found that age, non - DM without other comorbidities, and baseline VA score were found to be associated with VA improvement.

Multivariable models using propensity score weighting showed that there was a statistically significant difference in patients below 60 years of age for the PRN group, which increased the odds of improving vision by approximately three times compared to those over 60 years of age (odds ratio (OR) 2.92, 95% confidence interval (CI) = 1.4–6.07, *P*-value = 0.004 in patients < 50 years of age, and OR 3.25, 95%CI = 1.86–5.67, *P*-value = 0.000 in patients between 50 and 60 years of age). Furthermore, patients diagnosed with RVO were significantly associated with vision improvement of approximately three times higher than those of other diseases (95%CI = 1.20–6.34, *P*-value = 0.0170), and the odds of vision improvement per injection increased by 43% (95%CI = 14–78%, *P*-value = 0.0020). It was also found that patients who had lower vision at baseline were more likely to have improved VA than individuals with better vision at baseline (OR 1.08, 95%CI = 1.04–1.13, *P*-value = 0.000). However, there was no statistically significant difference between the IVB and IVR patients, sex or comorbidities. In the 3Inj pattern, patients less than 50 years of age, those between 50 and 60 years of age, and baseline VA were statistically significant predictors (*P*-value = 0.000, 0.006, and 0.009, respectively). Additionally, it was found that having DM without other comorbidities was also a statistically significant predictor of low response for vision improvement compared to DM with other comorbidities (OR 0.16, 95%CI = 0.03–0.76, *P*-value = 0.021).

**Table 4:** Multivariable analysis of vision improvement for the PRN pattern and three-injections pattern

Variables	PRN pattern				Three-injections pattern			
	Crude OR (95% CI)	P-value	Adjusted OR (95% CI)	P-value	Crude OR (95% CI)	P-value	Adjusted OR (95% CI)	P-value
Age								
>60 years	1.0 (reference)		1.0 (reference)		1.0 (reference)		1.0 (reference)	
50 to 60 years	1.18 (0.92-1.50)	0.189	3.25 (1.86-5.67)	0.000*	1.87 (1.00-3.51)	0.051	3.99 (1.48-10.76)	0.006*
<50 years	1.23 (0.95-1.58)	0.113	2.92 (1.4-6.07)	0.004*	2.24 (1.10-4.56)	0.027*	18.38 (4.61-73.36)	0.000*
Sex								
Male	1.0 (reference)		1.0 (reference)		1.0 (reference)		1.0 (reference)	
Female	1.03 (0.84-1.26)	0.788	0.8 (0.47-1.39)	0.434	1.34 (0.80-2.27)	0.271	1.20 (0.53-2.73)	0.668
Drug								
IVB	1.0 (reference)		1.0 (reference)		1.0 (reference)		1.0 (reference)	
IVR	1.20 (0.68-2.12)	0.691	1.18 (0.68-2.05)	0.561	0.48 (0.20-1.17)	0.109	0.86 (0.35-2.14)	0.753
Co-morbidity								
DM with other comorbidities	1.0 (reference)		1.0 (reference)		1.0 (reference)		1.0 (reference)	
DM without other comorbidities	1.27 (0.90-1.80)	0.179	1.02 (0.42-2.51)	0.961	0.41 (0.11-1.61)	0.202	0.16 (0.03-0.76)	0.021*
non-DM with other comorbidities	1.52 (1.15-2.01)	0.003*	0.91 (0.39-2.12)	0.819	1.31 (0.70-2.43)	0.400	0.83 (0.17-4.11)	0.817
non-DM without other comorbidities	1.55 (1.16-20.7)	0.003*	1.52 (0.63-3.65)	0.349	2.14 (1.06-4.32)	0.034*	0.70 (0.15-3.24)	0.645
Diagnosis								
Other diseases	1.0 (reference)		1.0 (reference)		1.0 (reference)		1.0 (reference)	
nAMD and PCV	1.57 (1.06-2.32)	0.023*	1.66 (0.73-3.78)	0.226	1.12 (0.48-2.63)	0.787	2.36 (0.52-10.7)	0.266
DME	1.32 (0.93-1.87)	0.126	1.39 (0.49-3.97)	0.535	1.21 (0.50-2.94)	0.675	0.88 (0.15-5.13)	0.883
RVO	3.66 (2.50-5.36)	0.000*	2.76 (1.20-6.34)	0.017*	2.26 (0.93-5.48)	0.072	2.39 (0.66-8.62)	0.182
PDR	1.25 (0.87-1.80)	0.223	0.85 (0.30-2.41)	0.756	0.88 (0.26-3.01)	0.835	0.42 (0.04-4.09)	0.454
Baseline VA^a^	1.05 (1.04-1.07)	0.000*	1.08 (1.04-1.13)	0.000*	1.08 (1.04-1.13)	0.001*	1.10 (1.02-1.18)	0.009*
Number of drug injections^a^	1.27 (1.06-1.54)	0.012*	1.43 (1.14-1.78)	0.002*	-	-	-	-

^a^Continuous variable

*P-value < 0.05

*OR* Odds Ratio, *95% CI* 95% confidence interval, *IVB* intravitreal bevacizumab, *IVR* intravitreal ranibizumab, *DM* diabetes mellitus, *nAMD* Neovascular age-related macular degeneration, *PCV* polypoidal choroidal vasculopathy, *DME* Diabetic macular edema, *RVO* Retinal vein occlusion, *PDR* Proliferative diabetic retinopathy, *VA* Visual acuity

## Discussion

To the best of our knowledge, this study demonstrated the first large cohort, prospective, observational IVB research for macular diseases using real-life outcome data from routine clinical practice in the Thai context. These findings showed that the proportion of visual response was not statistically different between the two drugs in both the PRN and 3Inj groups. It was also found that IVB was effective even in those with poor VA and was consistent with existing studies. For example, in nAMD treatments, patients who were treated with IVR recorded VA improvements equal to or greater than 15 ETDRS letters at a probability of approximately 33–41% [10, 11, 24, 25, 29]. In addition, results from the meta-analysis and network meta-analysis studies in AMD and DME treatments showed no significant difference between IVR and IVB [9, 12, 13, 30].

The results of this study also highlighted that in daily routine services, ophthalmologists used anti-VEGF drugs not only for nAMD and DME but also for other retinal diseases as well. In addition, most of the data revealed that the follow-up intervals were more than 35 days and that VA measurement methods used in subsequent visits were not the same as the baseline, e.g. the pinhole method was used during the first visit but another visit used a different method. As a result, this reduced the number of patients who were eligible for evaluating drug effectiveness to only 226 patients from the 3Inj group. To increase the number of samples for future studies, VA should be measured using the same method as the baseline within 35 days of each visit during the 6-month follow-up period.

Furthermore, since this was an observational study, eligible patients were diagnosed and treated by their own ophthalmologists; as such, decisions about drug prescriptions, switching treatments or stoppage of treatment were made by their own ophthalmologists. Consequently, the results showed that some demographic data at the baseline did not match the findings in some RCT studies. First, median patient age was lower than previous RCT studies (approximately 70 years and older for previous studies) [11, 29, 31, 32]. However, this study’s findings were consistent with research on AMD prevalence in Thailand [8], epidemiology of AMD among the Thai elderly population [33], and anti-VEGF drugs and its complications in Thailand [34]; these papers reported that the average age of their respective samples was approximately 60 years old. The reason for this may be that the median age of the patients in this study was lower as it included DME patients - who may have developed the disease at a younger age compared to nAMD patients or other retinal diseases but may also have a better prognosis as they received treatments earlier. Second, this study utilized the range of severity from NPL (unable to read the Snellen/ETDRS chart) until nearly normal VA for BCVA at baseline. However, the RCTs only included patients with BCVA from 20/25 to 20/320 [24, 29, 31, 32, 35–38]; this may reflect differences in the clinical setting, daily routine, and protocol of the controlled clinical trials. Therefore, median BCVA at baseline of the patients in this study may be better than some existing RCTs or worse than other studies.

In addition, VA measurement in the RCTs typically used ETDRS charts because it is the gold standard and is considerably superior to Snellen chart [39, 40]. However, this study found that several study sites performed routine ocular examinations using the Snellen chart since it was quick and simple to measure [39]. Moreover, the findings from Kalpana S. et al. [41] revealed that the Snellen and ETDRS charts may be used interchangeably for daily routine services or for RCTs. Regardless, this study used the equation proposed by Gregori et al. [26] to convert VA measured via the Snellen chart into approximate ETDRS letter scores.

When considering the number of injections, although the results showed that the proportion of VA response increased with the number of injections given – especially in the PRN pattern – it was seen that patients received a median of one injection for treatment during the 6-month follow-up period. This study’s results were similar to those from other non-interventional studies, which reported that the number of anti-VEGF injections in real-life conditions was considerably lower than in the RCTs [31, 42–45]. This could be due to other possible potential factors that were not accounted for in this study such as frequency of follow-up visits; prolonged duration of eye symptoms; not realizing the importance of treatment after receiving a good baseline VA; declining motivation to receive treatment after three consecutive doses; poor access to services and hospital facilities; and non-affordable travel expenses.

The multivariable model using propensity score weighting showed that age of less than 60 years and number of drug injections were found to be positive predictive factors of vision improvement; this is consistent with results from interventional and non-interventional studies [31, 32, 46]. In addition, many research papers, including review articles, indicated that higher BCVA scores at baseline were associated with less VA gain from baseline values [47–52]. A retrospective study that reviewed medical records also found that VA at baseline was the most significant predictor of VA outcomes in nAMD patients who received IVB for 6 months [53–56]. Patients diagnosed with RVO were a statistically significant predictor of response compared to other disorders, such as vitreous haemorrhage, only in the PRN group and represented the highest proportion of patients with improved VA scores. However, it was not possible to perform a multivariable logistic regression analysis for individual retinal disease – including RVO - due to the inadequate number of subjects. For the 3Inj group, DM without other comorbidities was a negative predictive factor compared to DM with other comorbidities; this may be limited by the type of study, number of samples, and other associated factors. Moreover, these favourable short-term results from the multivariable analysis suggests that further studies are needed in a larger group of patients with a longer follow-up period. For VA improvement between the IVB and IVR groups, the findings were consistent with other studies in that there was no significant difference between the two drugs [12, 13, 52].

### Policy implications in the Thai context

As mentioned in the introduction, the NLEM Sub-committee included bevacizumab into the NLEM to address the issues of anti-VEGF drug affordability and IVB treatment accessibility. However, even with the available international evidence from RCTs and observational studies, Thai policy-makers and the NLEM Sub-committee itself still required safety and effectiveness information to make the most informed decisions about this inclusion. For this reason, the NLEM Sub-committee requested for the development of a safety and effectiveness monitoring system and research on real-life practices. This contrasts with the more typical process used in other countries where trials are conducted to determine evidence on safety and efficacy evidence first prior to policy implementation. Therefore, the participants included in this study would be different compared to high-income countries, where most previous and similar studies were conducted.

Another factor which was taken into consideration was the Thai health insurance infrastructure. While there are three major public health insurance schemes in Thailand comprising the UCS, SSS, and CSMBS, only government officers under the CSMBS can reimburse IVR. In the past, retina specialists previously considered prescribing IVB first for intravitreal anti-VEGF therapies as it was already included in the NLEM. The reason for this was that cost was an important factor for prescribing drug since patients had to pay IVR costs out-of-pocket, and a single injection of IVR was more expensive than a single dose of IVB (1362 USD per injection for IVR versus 544 USD/vial for IVB - equivalent to 18.16 USD per injection) [57–59]. However, since the inclusion of bevacizumab into the NLEM for nAMD and DME in 2012, all Thai citizens now have extensive access to IVB free-of-charge and are able to experience similar VA improvement outcomes compared to IVR.

In addition, while this study was ongoing, the World Health Organization added bevacizumab to its Essential Medicine List for ophthalmic indication [60]. Hence, this action supports the use of IVB and its ability to create equitable access of essential medicines for macular disease patients in Thailand. Data from the national database also illustrated an increasing trend in the number of new patients who received IVB, rising from 2694 cases in fiscal year 2013 to 3908, 4535, and 6979 in fiscal years 2014, 2015, and 2016, respectively [61].

### Limitations of study

The results of this study should be interpreted with caution. First, the main study focused on generating evidence for IVB to inform policy-makers and the NLEM Sub-committee. Thus, this study primarily aimed to assess the effectiveness of IVB in real-life practice. Second, only a small number of patients received IVR. This imbalance in the number of patients and patient characteristics between the drug groups due to the lack of randomization may have led to bias and confounding but was addressed by using the propensity score method to minimise possible biases. Third, the follow-up period was only 6 months, and this may not have captured the long-term effect, efficacy or effectiveness of both drugs on visual outcomes. Although there are several RCT studies which utilized a follow-up period of 12-month, the results of studies conducted in LMICs are likely to be out of date as evidence generation in those real-world settings has been scarce. Therefore, capturing the long-term effect, efficacy, and effectiveness of both drugs is still important for these settings as they have different contexts from developed or high-income countries. Together with suggestions from Thai retina specialists, it is accepted that the follow-up period of 6 months is sufficient for real-world practice to examine the short-term effectiveness of anti-VEGF drugs in Thailand. However, the long-term outcomes such as 1-year monitoring and evaluation are still needed to confirm the effectiveness of this intervention for further feedback to stakeholders. Fourth, multiple VA measurement methods were used in real-world practice, including ETDRS and Snellen charts as well as semi-quantitative methods such as CF and HM; this contributed to the diversity in VA reporting among our study sites. Therefore, a formula [26] was used to convert all VA scores into the same unit for measuring outcomes. Lastly, unlike other studies, CMT measurement was not included in the analysis as it is not covered by the health schemes and is considered optional. When reviewing the medical records during the data gathering process, there were many cases of missing CMT data, making any further analysis unfeasible. Therefore, further studies should employ a matching case approach in the observational study to avoid selection bias and conduct a follow-up regarding the long-term consequences of IVB treatment. This should be conducted together with designing further policy implications for optimising treatment regimens and indications in the pharmaceutical benefit package.

## Conclusions

This study included patients with more varied diseases and demographics than in previous RCTs and used various treatment patterns which can be generalized to other real-life settings with macular patients. The results also provided evidence that under real-world conditions, IVB is just as effective as IVR in improving VA. Thus, IVB can be considered as an alternative drug for patients who need treatment but are unable to afford IVR.

The findings of this study may reflect real-world outcomes and generate evidence to inform policy for allocating resources, refining the NLEM, and providing information to ophthalmologists and patients to improve confidence in prescribing and using IVB. Additionally, the results may also benefit future policy implementation in the long-run for Thailand or other developing countries with similar interests by enhancing the retinal treatment system, safety monitoring systems, and individual data management systems for retinal diseases.

## Abbreviations

3Inj: Three-injections
BCVA: Best-corrected visual acuity
CATT: The Comparison of Age-related macular degeneration Treatments Trials
CF: Counting fingers
CI: Confidence interval
CKD: Chronic kidney disease
CMT: Central macular thickness
CSMBS: Civil Servant Medical Benefit Scheme
DLP: Dyslipidaemia
DM: Diabetes mellitus
DME: Diabetic macular edema
ETDRS: Early Treatment Diabetic Retinopathy Study
HITAP: Health Intervention and Technology Assessment Program
HM: Hand movements
HTN: Hypertension
IHD: Ischaemic heart disease
IQR: Interquartile ranges
IVAN: Inhibition of VEGF in Age-related choroidal Neovascularisation
IVB: Intravitreal bevacizumab
IVR: Intravitreal ranibizumab
nAMD: Neovascular age-related macular degeneration
NLEM: National list of essential medicines
NPL: No light perception
OR: Odds ratio
PCV: Polypoidal choroidal vasculopathy
PDR: Proliferative diabetic retinopathy
PL: Perception of light
PRN: Pro re nata
RCTs: Randomised control trials
RVO: Retinal vein occlusion
SSS: Social Security Scheme
UCS: Universal Coverage Scheme
VA: Visual acuity
VEGF: Vscular endothelial growth factor
